# SMEK1 ablation promotes glucose uptake and improves obesity-related metabolic dysfunction via AMPK signaling pathway

**DOI:** 10.1152/ajpendo.00387.2023

**Published:** 2024-04-03

**Authors:** Shijun Wei, Yu Song, Zhengbin Li, Ai Liu, Yunfang Xie, Shang Gao, Hongbiao Shi, Ping Sun, Zekun Wang, Yecheng Jin, Wenjie Sun, Xi Li, Jiangxia Li, Qiji Liu

**Affiliations:** ^1^Key Laboratory for Experimental Teratology of the Ministry of Education, Department of Medical Genetics, School of Basic Medical Sciences, Cheeloo College of Medicine, Shandong University, Jinan, People’s Republic of China; ^2^Key Laboratory for Experimental Teratology of the Ministry of Education, Department of Medical Genetics, School of Basic Medical Sciences, Shandong University, School of Health and Life Sciences University of Health and Rehabilitation Sciences, Qingdao, People’s Republic of China

**Keywords:** adipogenesis, AMP-activated protein kinase, glucose uptake, obesity, SMEK1

## Abstract

Obesity has become a major risk of global public health. SMEK1 is also known as a regulatory subunit of protein phosphatase 4 (PP4). Both PP4 and SMEK1 have been clarified in many metabolic functions, including the regulation of hepatic gluconeogenesis and glucose transporter gene expression in yeast. Whether SMEK1 participates in obesity and the broader metabolic role in mammals is unknown. Thus, we investigated the function of SMEK1 in white adipose tissue and glucose uptake. GWAS/GEPIA/GEO database was used to analyze the correlation between SMEK1 and metabolic phenotypes/lipid metabolism-related genes/obesity. *Smek1* KO mice were generated to identify the role of SMEK1 in obesity and glucose homeostasis. Cell culture and differentiation of stromal-vascular fractions (SVFs) and 3T3-L1 were used to determine the mechanism. 2-NBDG was used to measure the glucose uptake. Compound C was used to confirm the role of AMPK. We elucidated that SMEK1 was correlated with obesity and adipogenesis. *Smek1* deletion enhanced adipogenesis in both SVFs and 3T3-L1. *Smek1* KO protected mice from obesity and had protective effects on metabolic disorders, including insulin resistance and inflammation. *Smek1* KO mice had lower levels of fasting serum glucose. We found that SMEK1 ablation promoted glucose uptake by increasing p-AMPKα(T172) and the transcription of *Glut4* when the effect on AMPK-regulated glucose uptake was due to the PP4 catalytic subunits (PPP4C). Our findings reveal a novel role of SMEK1 in obesity and glucose homeostasis, providing a potential new therapeutic target for obesity and metabolic dysfunction.

**NEW & NOTEWORTHY** Our study clarified the relationship between SMEK1 and obesity for the first time and validated the conclusion in multiple ways by combining available data from public databases, human samples, and animal models. In addition, we clarified the role of SMEK1 in glucose uptake, providing an in-depth interpretation for the study of its function in glucose metabolism.

## Graphical abstract

**Figure:**
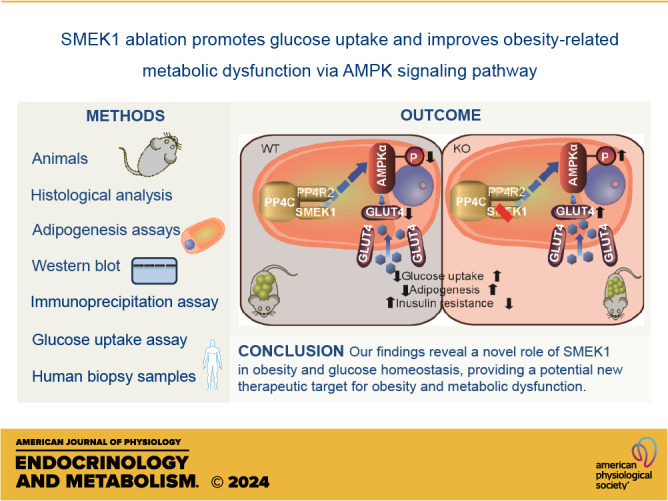

## INTRODUCTION

Obesity has become a major global public health challenge and is closely associated with cardiovascular and metabolic diseases, including type 2 diabetes, insulin resistance (IR), dyslipidemia, and nonalcoholic fatty liver disease (NAFLD) (1, 2). Obesity is characterized by excessive expansion of white adipose tissue (WAT). WAT plays a key regulatory role in the energy homeostasis of the system. Under the pressure of overnutrition, WAT expands by increasing the size of adipocytes (hypertrophy) or promoting adipocyte differentiation to generate new adipocytes during adipogenesis (hyperplasia). The hypertrophy of adipocytes is maladaptive and associated with pathological WAT remodeling, whereas proliferation is generally considered to be adaptive and conducive to metabolic health (3, 4).

AMP-activated protein kinase (AMPK) is a serine/threonine-specific protein kinase that exists as a multiple heterotrimeric complex comprised of a catalytic α-subunit (α1 and α2) and regulatory β (β1 and β2) and γ (γ1, γ2, γ3) subunits (5). AMPK acts as an energy sensor in cellular metabolic homeostasis. AMPK activity is regulated in response to stresses that elevate the cellular AMP/ATP ratio (6). In addition to allosteric activation by AMP, AMPK is activated through the phosphorylation of a critically self-contained threonine residue (Thr172) by upstream kinases (5, 7). AMPK activation enhances transcription of glucose transporter 4 (GLUT4) and leads to GLUT4 translocation from the cytosol to the plasma membrane (7).

Suppressor of MEK1 (SMEK1), also known as a regulatory subunit of protein phosphatase 4 (PP4). PP4 is a protein complex composed of a catalytic subunit PP4c and regulatory subunits (8). Several PP4-specific regulatory subunits have been identified, including PP4R1, PP4R2, PP4R3, PP4R3B, and PP4R4. Human PP4R3 exists in two isoforms: PP4R3A (SMEK1) and PP4R3B (SMEK2), both regulatory subunits are shown to form complexes with PP4c-PP4R2, the major form of the holoenzyme PP4 (9). PP4 participates in many physiological and pathological processes. Mitochondrial Ca^2+^ uniporter (MCU)-mediated Ca^2+^ uptake perturbs lipid metabolism via PP4-dependent AMPK dephosphorylation (10). PP4 also functions as a key regulator of tumor necrosis factor (TNF)-α-induced insulin resistance in liver (11).

Studies have documented the importance of the interaction of different regulatory subunits with PP4c in controlling the activity of the holoenzyme (12). SMEK1 regulates the activity of PP4 catalytic subunits, but the mechanism is still unknown (9, 13). Moreover, SMEK1 has been shown to have many functions, some of which are dependent on PP4, while others are not. Relating to metabolic function, SMEK1 was first reported to play a role in metabolic homeostasis as a key regulator of hepatic gluconeogenesis in 2010 (14). In 2014, Pph3-Psy2, the yeast counterpart of the mammalian PP4c-R3 complex, was reported to dephosphorylate glucose transporter genes (15). Recently, Smek1 was identified as a dual-function regulator of lipid and carbohydrate metabolism during Magnaporthe infection in rice (16).

Nevertheless, the metabolic function of SMEK1 in mammals has not been extensively clarified to date. Previous genome-wide association studies (GWAS) identified that SNPs of SMEK1 are correlated with metabolic phenotypes (https://www.gwascentral.org/). In this study, we proposed that SMEK1 might participate in the pathogenesis of obesity and metabolic regulation. Results in this study showed that SMEK1 inhibited adipogenesis of preadipocytes. Using *Smek1* KO mice, we investigated the role of SMEK1 in obesity and glucose homeostasis. Our findings reveal the role of SMEK1 in lipid and glucose metabolism and provide a potential new therapeutic target for obesity and related diseases.

## MATERIALS AND METHODS

### Animals

All animal experiments were performed in accordance with protocols approved by the Institutional Animal Care and Use Committee, School of Basic Medical Sciences, Shandong University. *Smek1* global knockout (*Smek1* KO) mice on a C57BL/6 background have been previously described (17). In brief, *Smek1^fl/fl^* mice were generated by delivering a linearized vector to ES cells by Cyagen Biosciences. *Smek1* KO mice were obtained by mating *Smek1^fl/fl^* mice with *Sox2*-Cre mice. Mouse genotyping was performed by polymerase chain reaction (PCR) as shown in Supplemental Fig. S1, and the sequences of primers are listed in Supplemental Table S1. Ablation of SMEK1 is also analyzed by Western blot analysis. In our study, we obtained overexpressed *Smek1*-HA in adipose tissue of mice by crossbreeding *ROSA26*-*Smek1^flox/flox^* mice (Biocytogen Corporation, Beijing, China) with *Adipoq*-Cre mice (Cyagen Biosciences Inc., Guangzhou, China). The mice were housed in pathogen-free facility in plastic cages at 22–24°C, 40–50% humidity, with a 12-h daylight cycle from 6:00 AM to 6:00 PM. Unless otherwise specified, the mice had free access to drink and water. Except for stromal-vascular fraction (SVF) isolation, mice designed for the animal experiments were all male mice aged 3–4 mo.

### Body Composition and Energy Expenditure

Body composition of mice was measured using NMR (Bruker Scientific, LF90II). Oxygen consumption (V̇o_2_), carbon dioxide production (V̇co_2_), and heat production were determined by using a Comprehensive Lab Animal Monitoring System (Columbus Instruments, CLAMS-16).

### GTT and ITT

#### Glucose tolerance test.

WT and *Smek1* KO mice were starved for 6 h and free to water. Glucose (Sigma-Aldrich, D9434, 1.5 g/kg) was injected intraperitoneally, and blood glucose was monitored at the indicated time points (0, 15, 30, 45, 60, 90 min) with glucometer (Roche, ACCU-CHEK Active).

#### Insulin tolerance test.

WT and *Smek1* KO mice were starved for 6 h and free to water. Insulin (Beyotime Biotechnology, Shanghai, China, P3376, 0.75 units/kg) was injected intraperitoneally, and blood glucose was monitored at the indicated time points (0, 15, 30, 45, 60, 90 min) with a glucometer (Roche, ACCU-CHEK Active).

#### Pyruvate tolerance test.

WT and *Smek1* KO mice were starved for 16 h and free to water. Sodium pyruvate (BBI, Shanghai, China, A600884, 2 g/kg) was injected intraperitoneally, and blood glucose was monitored at the indicated time points (0, 15, 30, 45, 60, 90 min) with a glucometer (Roche, ACCU-CHEK Active).

### Metabolic Studies

Mice were fasted overnight (12 h), and tail vein blood was collected. Serum samples were stored at −80°C until use. Blood glucose was measured as described earlier. Concentrations of insulin (Beyotime Biotechnology, China, PI602), glucagon (Beyotime Biotechnology, China, PG357), leptin (Solarbio Life Sciences, Beijing, China, SEKM-0105), IL-6 (Dakewe Biotech, China, 1210602), norepinephrine (Nanjing Jiancheng Bioengineering Institute, Nanjing, China, H096-1-2), and epinephrine (Nanjing Jiancheng Bioengineering Institute, Nanjing, China, H208-1-2) were measured using ELISA kits. Concentrations of triglycerides (Nanjing Jiancheng Bioengineering Institute, Nanjing, China, A110-1-1), total cholesterol (Nanjing Jiancheng Bioengineering Institute, Nanjing, China, A111-1-1), HDL (Nanjing Jiancheng Bioengineering Institute, Nanjing, China, A112-1-1), LDL (Nanjing Jiancheng Bioengineering Institute, Nanjing, China, A113-1-1), and free fatty acid (Nanjing Jiancheng Bioengineering Institute, Nanjing, China, A042-2-1) were measured using assay kits.

### Histological Analysis

The adipose tissues were collected in 4% paraformaldehyde (Servicebio, Wuhan, China, G1101) and incubated for at least 48 h. Paraffin-embedded adipose tissues were cut into 5-mm-thick sections, adhered onto glass slides, deparaffinized by dimethylbenzene, and rehydrated by decreasing ethanol concentrations.

#### H&E staining.

Paraffin-embedded sections were stained with hematoxylin (Servicebio, Wuhan, China, G1004) and eosin (Servicebio, G1002) as described and imaged with microscope (KEYENCE, BZ-X800). Adipocyte areas were calculated with Image J.

#### Immunofluorescence.

Rehydrated tissues were boiled in antigen repair buffer (ZSGB-Bio, Beijing, China, ZLI-9069) and blocked with 4% serum for 45 min at room temperature, then incubated with primary antibodies against SMEK1 (Abclonal Technology, A8500) overnight at 4°C. The next day, we incubated with donkey anti-rabbit IgG (Alexa Fluor594, Abcam, ab150076). The slides were sealed with DAPI (Abcam, ab104139), and imaged with microscope.

#### Immunohistochemistry.

The previous operation of rehydrated tissues was the same as immunofluorescence (IF). The next day, we incubated with horseradish peroxidase-labeled secondary antibodies (Jackson ImmunoResearch, 111-035-003). The slides were sealed with rhamsan gum (Servicebio, Wuhan, China, WG10004160) and then imaged with microscope.

### Isolation of Stromal-Vascular Fraction and Adipogenesis Assays

SVFs were isolated from inguinal adipose tissue as previously described (18). Briefly, iWAT was dissected from mice aged 4–6 wk, rinsed in PBS, minced, and digested for 40–50 min at 37°C in 2 mg/mL type I collagenase solution (Diamond, Shanghai, China, A004194) with PBS. Digested tissue was centrifuged at 700 *g* for 10 min and filtered through a 70 μm cell filter. The sediment was collected by centrifugation again. SVFs were cultured in DMEM/F12 (Procell Life Science & Technology, Wuhan, China, PM150312) with 10% fetal bovine serum (FBS, Sigma-Aldrich, F0193) added in penicillin (BBI, Shanghai, China, A613460) and streptomycin (BBI, Shanghai, China, A610494). During adipogenic differentiation, SVFs or 3T3-L1 cells were induced with differentiation media containing DMEM/F12 with 10% FBS, 0.1%penicillin, 0.1%streptomycin, 0.5 mM 3-isobutyl-1-methylxanthine (IBMX, Sigma-Aldrich, I5879), 2 μg/mL dexamethasone (BBI, Shanghai, China, A601187), 5 μg/mL insulin for 3 days and then cultured in induction medium containing DMEM/F12 with 10% FBS, penicillin, streptomycin, and insulin for another 6 days.

### Cell Culture

3T3-L1 preadipocytes (CL-173, ATCC, sex unknown) were maintained in the dedicated medium (Procell Life Science & Technology, Wuhan, China, CM-0006). Cells were grown at 37°C, 5% CO2 humid atmosphere. The *SMEK1* expression vector was constructed using the full length of human *SMEK1* coding sequence (ENSG00000100796.17), then cloned into the pLVX-IRES-puro vector. The vector of pLVX-IRES-puro or pLVX-SMEK1 was used to infect 3T3-L1 cells by lentivirus, leading to the generation of *SMEK1* overexpressing and control 3T3-L1 cell lines. Similarly, the *PPP4C* expression vector was constructed using the full length of human *PPP4C* coding sequence (NM_001303503.1), leading to the generation of *PPP4C* overexpressing and control SVFs. The GV248 lentiviral vector containing sh*Smek1* was used for the generation of *Smek1* KD 3T3-L1 cells. pLKO.1-EGFP-puro expressed sh*Ppp4c* was used for the generation of *Ppp4c* KD SVFs. In the AMPK inhibitor Compound C (CC, also known as Dorsomorphin, MCE, HY-13418A) experiments, adipocytes were pretreated with 10 µM CC for 1 h (19).

### RNA Isolation and qPCR

Total RNA was extracted from tissues or cells using TRIzol reagent (Vazyme, Nanjing, China, R401-01) and reverse transcribed into cDNA with HiScript III RT SuperMix for qPCR (Vazyme, Nanjing, China, R323-01). Quantitative PCR was performed in 20 μL of the brilliant SYBR green PCR master mixture (CWBio, Jiangsu, China, CW0957H) using a real-time PCR System (Roche, LightCycler480). The expression levels of mRNA were calculated as relative fold changes by the 2−^ΔΔCT^ method and normalized to *GAPDH* or *18S*. Sequences of qPCR primers are listed in Supplemental Table S2.

### Western Blot Analysis

Total protein samples were isolated by treating tissue or cell samples with RIPA lysis buffer (CWBio, Jiangsu, China, CW2333S) and phosphatase inhibitor cocktail (APExBio, K1015, K1007). The BCA Protein Assay kit (Real-gen Biotechnology, Jiangsu, China, K10007A) was used to measure protein concentrations. Proteins were separated using 10% SDS-PAGE gels and then transferred to PVDF membranes (GVS, 1212639). After the membranes were blocked in 5% skim milk, they were incubated overnight at 4°C with primary antibodies and then for 1 h at room temperature with the corresponding secondary antibodies. A ChemiDoc MP Imaging System (Cytiva, Amersham Imager 600) was used for signal detection. Protein expression levels were quantified using ImageJ software and normalized to the levels of HSP90. Details of the antibody are listed in Supplemental Table S3.

### Immunoprecipitation Assay

Differentiated SVFs isolated from *Smek1*-HA mice were lysed in ice-cold immunoprecipitation (IP) buffer (50 mM Tris pH 7.5, 100 mM NaCl, 2 mM EDTA PH8.0, 1% NP40 and 2 mM PMSF) and centrifuged at 12,000 rpm for 10 min. The cell lysates were then incubated with the indicated HA-tag antibody (Proteintech, Wuhan, China, 51064-2-AP) and protein A/G magnetic beads (MCE, HY-K0202) for 6 h at 4°C followed by washing in cold IP buffer. The normal mouse IgG (Santa Cruz Biotechnology, sc-2025) was used as a negative control. The immunocomplexes were collected and subjected to immunoblotting.

### Oil Red O Staining

First, we prepared a fresh Oil Red O working solution by adding 600 μL Oil Red O staining solution (5 mg/mL, BBI, Shanghai, China, A600395) to 400 μL distilled water. And then filtered the mixture through a 0.45 μm filter (Millipore, SLHV033RB). We then removed the culture media completely and rinsed the cells with PBS. The cells were fixed with 4% paraformaldehydes for 30 min at room temperature. After fixing, we gently rinsed the cells twice with PBS and added the Oil Red O working solution, and then incubated for 60 min at room temperature. Finally, we removed the staining solution and washed the cells with PBS two times. The red-stained lipid droplets were imaged with microscope.

### Glucose Uptake Assay

Glucose uptake by SVFs was assessed using 2-NBDG as previously described (20). Briefly, differentiated SVFs isolated from WT or KO mice were incubated with 2-NBDG (50 µM, MCE, HY-116215) for 30 min at 37°C. The adipocytes were washed with PBS three times for 5 min each, and fluorescence was observed and imaged with microscope.

### Measurement of GLUT4 Translocation

We fixed differentiated SVFs with 4% PFA for 30 min and then blocked with 4% serum as previously described. Next, incubated with primary antibodies against GLUT4 overnight at 4°C. The next day, we incubated with donkey anti-mouse IgG (Alexa Fluor594, Abcam, ab150108). Finally, the slides were sealed with DAPI (Abcam, ab104139) and imaged with microscope.

Cell membranes and cytoplasmic proteins of differentiated SVFs were separated using the Membrane and Cytosol Protein Extraction kit (Beyotime Biotechnology, Shanghai, China, P0033). The level of GLUT4 in the membrane was analyzed via Western blot.

### Human Subcutaneous Adipose Tissue

Biopsy samples of subcutaneous adipose tissue were obtained from 15 Chinese people receiving elective surgery in Qilu Hospital of Shandong University. Body mass index was calculated by height (in m) and weight (in kg). The age of the subjects ranged from 21 to 38 yr, and all subjects had a BMI between 28.4 and 64.57 kg/m^2^. The study received ethical clearance from Qilu Hospital of Shandong University.

### Database Search in GWAS Central and GEPIA

**Figure 1.**
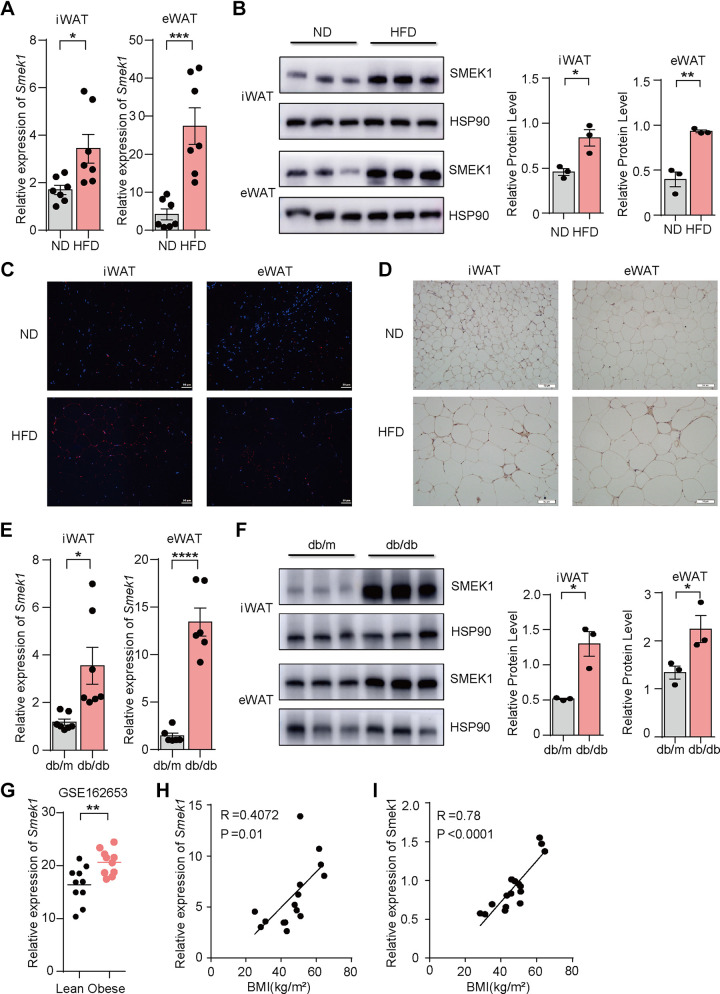
SMEK1 is associated with obesity. *A*: *SMEK1* mRNA levels in white adipose tissue of HFD and control mice (*n* = 7). *B*: SMEK1 protein levels in white adipose tissue of HFD and control mice. *C*: immunofluorescence staining of SMEK1 in white adipose tissue of HFD and control mice. Scale bar, 50 μm. *D*: immunohistochemical staining of SMEK1 in white adipose tissue of HFD and control mice. Scale bar, 50 μm. *E*: *SMEK1* mRNA levels in white adipose tissue of db/db and control mice(*n*=7). *F*: *SMEK1* protein levels in white adipose tissue of db/db and control mice. *G*: *SMEK1* mRNA levels in human white adipose tissues from GSE162653 (*n* = 10, two-sided *t* test). *H*: correlation analysis of *SMEK1* mRNA level and BMI in human adipose tissue (*n* = 15). *I*: correlation analysis of *SMEK1* protein level and BMI in human adipose tissue (*n* = 15) (ns means no significance, *P < 0.05, **P < 0.01, ***P < 0.001, ****P < 0.0001). BMI, body mass index; HFD. high-fat diet.

The human sequencing data of RNA-seq data reused in Fig. 1*F* of this study are available in Gene Expression Omnibus (GEO database) under accession code GSE162653. We searched the correlation of SMEK1 and related genes in the GEPIA database.

### Statistical Analysis

Data were analyzed by Prism 8.0 software (GraphPad) with unpaired two-tailed Student’s *t* tests or one-way ANOVA. Data were presented as means ± SE or means ± SD. Differences were considered significant when *P* < 0.05. *n* represents biological replicates.

## RESULTS

### SNPs in SMEK1 Are Correlated with Metabolic Phenotypes

Although SMEK1 participates in many physiological and pathological processes, its role in obesity and metabolic diseases remains unclear. Thus, we searched SNPs located in *SMEK1* in GWAS CENTRAL (https://www.gwascentral.org/) first and found most of the SNPs correlated with metabolic phenotypes. As summarized in Table 1, dozens of SNPs had a significant correlation with fasting plasma glucose, including rs876561, rs2273674, rs10134560, rs10498627, rs11628439, rs5020186, rs7154224, and rs997169. SNPs including rs2273674, rs4904785, rs12888580, rs8015483 are correlated with 2-h glucose challenge. In addition, three SNPs (rs876561, rs8010382, rs997169) are correlated with type II diabetes. SNPs of *SMEK1* are also correlated with birth weight (rs2180886) and other processes related to diabetes (rs2180886, rs10134560). And rs2025066 had a significant correlation with both insulin resistance and fasting insulin (Table 1). These results implied a potential clinical relevance of SMEK1 to obesity and metabolic phenotypes, especially glucose homeostasis and insulin homeostasis in the human population.

**Table 1.** SNPs of SMEK1 correlated with metabolic disorders

Phenotype	SNP	*P* Value	Alleles	Position	Dataset Identifier
Fasting plasma glucose	rs876561	0.01324	C > T	chr14:91944911	HGVRS3269
Fasting plasma glucose	rs2273674	0.000165	C > T	chr14:91928286	HGVRS3269
Fasting plasma glucose	rs10134560	0.008027	C > T	chr14:91930152	HGVRS3269
Fasting plasma glucose	rs10498627	0.007477	A > G	chr14:91972119	HGVRS3269
Fasting plasma glucose	rs11628439	0.01317	C > T	chr14:91946995	HGVRS3269
Fasting plasma glucose	rs5020186	0.008059	T > G	chr14:91928992	HGVRS3269
Fasting plasma glucose	rs7154224	0.007458	C > A	chr14:91964874	HGVRS3269
Fasting plasma glucose	rs997169	6.26E-03	C > T	chr14:91934303	HGVRS3269
Two-hour glucose challenge	rs2273674	0.05	C > T	chr14:91928286	HGVRS3278
Two-hour glucose challenge	rs4904785	0.009862	C > T	chr14:91956870	HGVRS3278
Two-hour glucose challenge	rs12888580	0.01245	A > G	chr14:91930824	HGVRS3278
Two-hour glucose challenge	rs8015483	9.95E-03	A > G	chr14:91955729	HGVRS3278
Type II diabetes	rs876561	0,02212	C > T	chr14:91944911	HGVRS9
Type II diabetes	rs8010382	7.00E-09	A > G	chr14:91963722	HGVRS9404
Type II diabetes	rs8010382	3.00E-12	A > G	chr14:91963722	HGVRS15031
Type II diabetes	rs8010382	6.00E-12	A > G	chr14:91963722	HGVRS15035
Type II diabetes	rs997169	4.06E-02	C > T	chr14:91934303	HGVRS9
Decline in glucose metabolism in posterior cingulate cortex	rs2273674	4.00E-08	C > T	chr14:91928286	HGVRS7884
Birth weight	rs2180886	0.003422	C > T	chr14:91972696	HGVRS590
Homeosatic model assessment of insulin resistance	rs2025066	0.04256	C > A	chr14:91958862	HGVRS3268
Fasting insulin	rs2025066	0.04257	C > A	chr14:91958862	HGVRS3266
Diabetic nephropathy in type I diabetes	rs2180886	0.042753	C > T	chr14:91972696	HGVRS1729
Medication use (drugs used in diabetes)	rs10134560	5.00E-08	C > T	chr14:91930152	HGVRS14087

### SMEK1 is Associated with Obesity

To investigate the metabolic function of SMEK1, first, we explored whether SMEK1 is associated with obesity. We induced obesity in a mice model by feeding them a high-fat diet for 12 wk and observed that both mRNA and protein expression levels of *Smek1* significantly increased in the white adipose tissue of obese mice compared with mice fed a normal diet (Fig. 1, *A* and *B*). We performed immunofluorescence staining (Fig. 1*C*) and immunohistochemistry staining (Fig. 1*D*) to detect the expression of SMEK1 in the white adipose tissue of obese mice and control mice. We observed that the expression of SMEK1 increased in obese mice. And db/db mice (leptin receptor mutant) are a common animal model that develops metabolic diseases such as obesity and insulin resistance in adulthood. We found that the mRNA and protein expression levels of *Smek1* significantly upregulated in both inguinal adipose tissue (iWAT) and epididymal adipose tissue (eWAT) in db/db mice compared with control mice (Fig. 1, *E* and *F*). In addition, we found that published data in the Gene Expression Omnibus (GEO) database (GSE162653) revealed increased *SMEK1* mRNA expression in WAT of individuals with obesity compared with lean individuals (Fig. 1*G*). Similarly, we collected human adipose tissue samples and found that the mRNA and protein expression levels of *SMEK1* positively correlated with BMI (Fig. 1, *H* and *I*). Together, the positive correlation of SMEK1 expression in WAT with obesity implies an important role of this protein in adipose tissue metabolism.

### SMEK1 Inhibits Adipogenesis In Vitro and In Vivo

**Figure 2.**
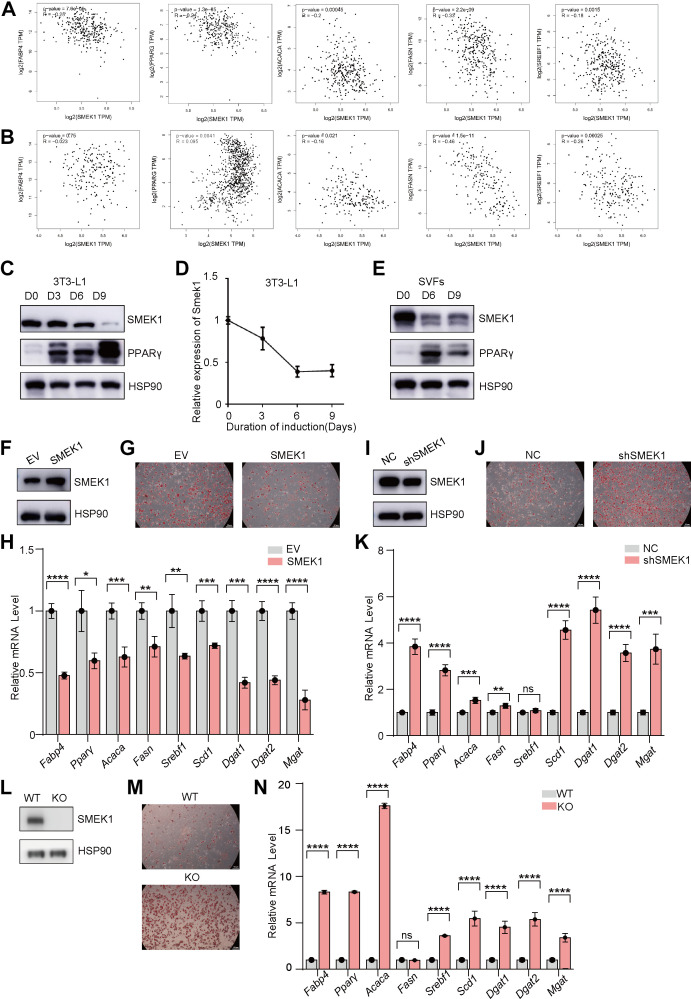
SMEK1 inhibits adipogenesis in vitro. *A*: correlation between SMEK1 and adipogenesis genes in subcutaneous adipose tissue in the GEPIA database. *B*: correlation between SMEK1 and adipogenesis genes in epididymal adipose tissue in the GEPIA database. *C*: SMEK1 protein expression during 3T3-L1 cells differentiation. *D*: *Smek1* mRNA expression during 3T3-L1 cell differentiation (*n* = 7). *E*: SMEK1 protein expression during SVFs differentiation. *F*: SMEK1 protein expression in control and *Smek1* OE 3T3-L1 cells. *G*: oil Red O staining of *Smek1* OE and control 3T3-L1 cells. *H*: the mRNA levels of adipogenesis genes of *Smek1* OE and control 3T3-L1 cells (*n* = 7). *I*: SMEK1 protein expression in control and *Smek1* KD 3T3-L1 cells. *J*: oil Red O staining of *Smek1* KD and control 3T3-L1 cells. *K*: the mRNA levels of adipogenesis genes of *Smek1* KD and control 3T3-L1 cells (*n* = 7). *L*: SMEK1 protein expression of SVFs isolated from WT and KO mice. *M*: oil Red O staining of SVFs isolated from WT and KO mice. *N*: the mRNA levels of adipogenesis genes of SVFs isolated from WT and KO mice (*n* = 7) (ns means no significance, *P < 0.05, **P < 0.01, ***P < 0.001, ****P < 0.0001). KD, knockdown; KO, knockout; SVFs, stromal-vascular fractions; WT, wild type.

We further determined the role of SMEK1 in adipogenesis. By searching for a correlation of the expression of SMEK1 with adipogenesis-related genes in the GEPIA database, we found a negative correlation between the expression of SMEK1 and adipogenesis-related genes, including *FABP4*, *PPARG*, *ACACA*, *FASN*, and *SREBF1* in both iWAT (Fig. 2*A*) and eWAT (Fig. 2*B*), especially in iWAT. Then we confirmed the function of SMEK1 in adipogenic differentiation. The *Smek1* protein and mRNA expression were reduced during 3T3-L1 adipogenic differentiation (Fig. 2, *C* and *D*). We isolated SVFs from 6-wk-old wild type (WT) mice, similarly, the SMEK1 protein reduced during SVFs adipogenic differentiation (Fig. 2*E*).

We used lentiviral infection to construct *Smek1* overexpression (OE, Fig. 2*F,* Fig. S2*C*) or knockdown (KD; Fig. 2*I*, Fig. S2*D*) cell lines. We assessed the samples on *day 9* of induction by Oil Red O staining and analyzed the mRNA levels of adipogenesis genes of *Smek1* OE or KD 3T3-L1 cells and their control group by real-time quantitative PCR. Oil Red O staining shows fewer lipid drops in *Smek1* OE 3T3-L1 cells (Fig. 2*G*). On the contrary, *Smek1* KD 3T3-L1cells have more lipid drops (Fig. 2*J*). We also found that the mRNA levels of adipogenesis genes, including *Fabp4*, *Ppar*γ, *Acaca*, *Fasn*, *Srebf1*, *Scd1*, *Dgat1*, *Dgat2*, *and Mgat*, were downregulated in *Smek1* OE 3T3-L1 cells (Fig. 2*H*). In contrast, these genes were upregulated in *Smek1* KD 3T3-L1 cells (Fig. 2*K*). Then we isolated SVFs from 6-wk-old WT and KO mice (Fig. 2*L*). Oil Red O staining showed more lipid drops in *Smek1* KO SVFs (Fig. 2*M*, Fig. S2*E*), adipogenesis-related genes were also upregulated (Fig. 2*N*). Overall, these data in vitro and vivo indicated that SMEK1 inhibits adipogenesis.

### Smek1 KO Mice Are Less Prone to Obesity and Related Metabolic Dysfunction

**Figure 3.**
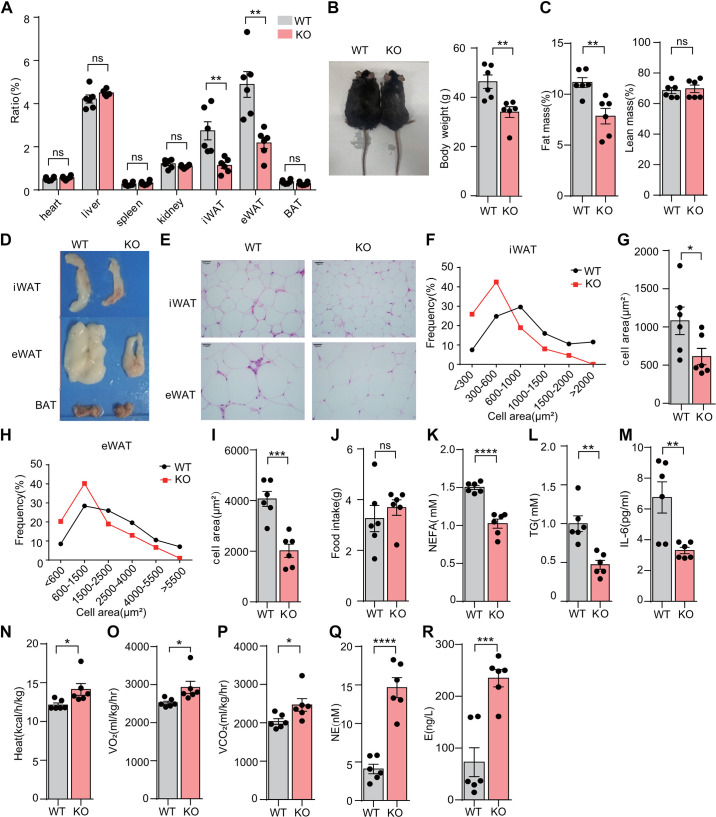
*Smek1* KO mice are less prone to obesity and related metabolic dysfunction (*n* = 6). *A*: organs and tissues ratio of WT and KO mice. *B*: gross view and body weight of WT and KO mice. *C*: fat mass and lean mass of WT and KO mice. *D*: gross view in adipose tissue of WT and KO mice. *E*: H&E staining in white adipose tissue of WT and KO mice, Scale bar, 10 μm. *F*: frequency distribution of inguinal adipocytes size in WT and KO mice. *G*: the average area of inguinal adipocytes between WT and KO mice. *H*: frequency distribution of epididymal adipocytes size in WT and KO mice. *I*: the average area of epididymal adipocytes between WT and KO mice. *J*: food intake of WT and KO mice. *K*: the NEFA content in the serum of WT and KO mice. *L*: the TG content in the serum of WT and KO mice. *M*: the IL-6 content in the serum of WT and KO mice. *N*: heat production of WT and KO mice. *O*: oxygen consumption of WT and KO mice. *P*: carbon dioxide production of WT and KO mice. *Q*: the norepinephrine content in the serum of WT and KO mice. *R*: the epinephrine content in the serum of WT and KO mice (ns means no significance, *P < 0.05, **P < 0.01, ***P < 0.001, ****P < 0.0001). KO, knockout; WT, wild type.

To investigate the specific role of SMEK1 in vivo, we generated *Smek1* KO mice using the loxp-cre system. As we expected, Western blot analysis revealed a significant reduction in SMEK1 expression in iWAT and eWAT of *Smek1* KO mice compared with WT mice (Supplemental Fig. S1). First, we measured the weight of tissues and organs, including heart, liver, spleen, kidney, iWAT, eWAT, and BAT, and then calculated the ratio to body weight in mice between two genotypes. The ratio of white adipose tissue was significantly reduced in KO mice compared with WT mice, while the ratio of other organs and tissues was unchanged (Fig. 3*A*). KO mice are lean and have lower body weight compared with WT mice (Fig. 3*B*).

We stated body composition by NMR. The fat mass of KO mice was significantly reduced compared with WT littermates, while the lean mass was unchanged (Fig. 3*C*). As expected, the volume of iWAT and eWAT in KO mice is smaller than in WT mice, suggesting that decreased body weight of KO mice is due to the difference of fat (Fig. 3*D*). Consistent with the inhibitory role of SMEK1 in adipogenesis, H&E staining showed that the iWAT and eWAT from KO mice had larger number and smaller adipocyte size than their control littermates (Fig. 3*E*). We counted the cell area of 600 adipocytes from a total of 30 fields taken from different sections of white adipose tissue in two different genotype mice. The frequency distribution curve of adipocyte area was plotted. As expected, the adipocytes of iWAT (Fig. 3, *F* and *G*) and eWAT (Fig. 3, *H* and *I*) from KO mice had a smaller size than WT mice. Although the food intake was unchanged between the two genotypes (Fig. 3*J*), the circulating levels of NEFA, TG, and IL-6 in KO mice were lower than WT (Fig. 3, *K–M*). The levels of HDL and LDL were also unchanged (Fig. S3).

We evaluated the effect of SMEK1 ablation on basic metabolic activity. KO mice showed significantly enhanced oxygen consumption, carbon dioxide production, and heat production compared with controls (Fig. 3, *N–P*). We also checked the levels of catecholamines. The contents of norepinephrine (NE) and epinephrine (E) were increased in the serum of KO mice (Fig. 3, *Q* and *R*). Overall, the enhanced metabolic rate might account for the lower body weight of KO mice.

### Smek1 KO Mice Enhance Insulin Sensitivity and Glucose Uptake

**Figure 4.**
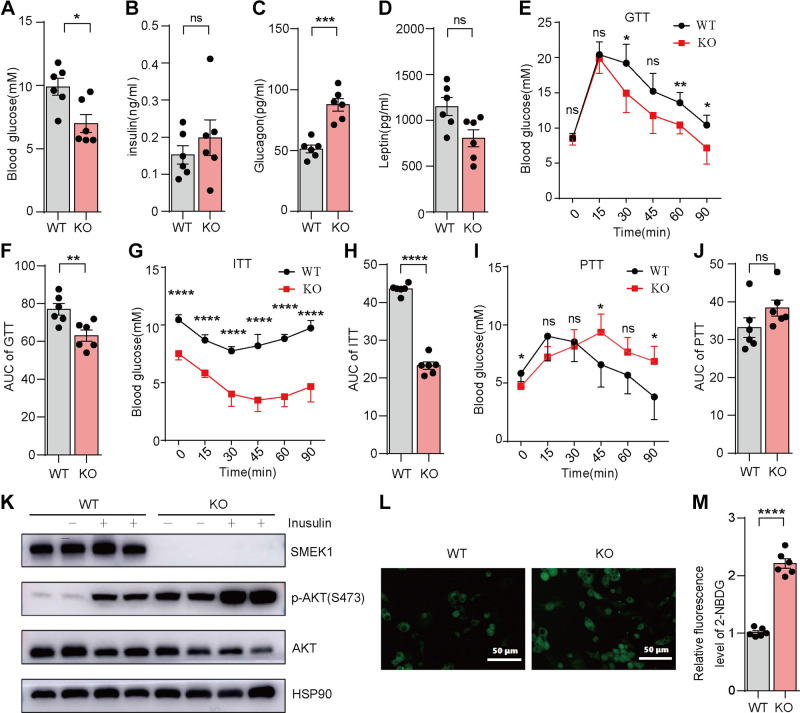
*Smek1* KO mice enhance insulin sensitivity and glucose uptake (*n* = 6). *A*: fasting blood glucose levels of WT and KO mice. *B*: fasting insulin levels in the serum of WT and KO mice. *C*: fasting glucagon levels in the serum of WT and KO mice. *D*: the Leptin content in the serum of WT and KO mice. *E*: glucose tolerance test of WT and KO mice. *F*: area under the curve of glucose tolerance test. *G*: insulin tolerance test of WT and KO mice. *H*: area under the curve of insulin tolerance test. *I*: pyruvate tolerance test of WT and KO mice. *J*: area under the curve of pyruvate tolerance test. *K*: analysis of insulin-stimulated p-AKT(S473) in epididymal adipose tissue. *L*: 2-NBDG glucose uptake experiment of SVFs isolated from WT and KO mice. Scale bar, 50 μm. *M*: quantification of luminosity in glucose uptake experiments (ns means no significance, *P < 0.05, **P < 0.01, ***P < 0.001, ****P < 0.0001). KO, knockout; SVFs, stromal-vascular fractions; WT, wild type.

Obesity often results in systemic glucose intolerance and insulin resistance; therefore, we assessed glucose homeostasis in the NCD-fed WT and KO mice. The level of fasting blood glucose level was lower in the serum of *Smek1* KO mice(Fig. 4*A*). We also found increased level of glucagon in KO mice (Fig. 4*C*). There was no change in insulin or leptin levels between the two genotypes (Fig. 4, *B* and *D*). Moreover, the *Smek1* KO mice showed improved glucose and insulin tolerance compared with their littermates, as shown in glucose tolerance test (GTT; Fig. 3*E*) and insulin tolerance test (ITT; Fig. 3*G*). Then we calculated AUC (area under the curve) to evaluate the results, which also visually illustrates the above conclusions (Fig. 4, *F* and *H*). We also did pyruvate tolerance test (PTT) with the same set of mice after starving for 16 h to evaluate gluconeogenesis. Although the level of fasting blood glucose level was lower in KO mice, they have enhanced ability of gluconeogenesis (Fig. 4, *I* and *J*). To test whether deletion of SMEK1 affects insulin-stimulated p-AKT (S473) in AKT signaling, 2-mo-old mice were fasted for 4 h and injected with 2 units/kg insulin. eWAT was isolated for 10 min after insulin injection. Western blot analysis showed increased p-AKT (S473) in eWAT of *Smek1* KO mice compared with WT mice (Fig. 4*K*).

Considering the role of SMEK1 in glucose homeostasis and insulin sensitivity, we investigated whether SMEK1 affects glucose uptake. We isolated SVFs from iWAT of WT or KO mice, then induced differentiation into mature adipocytes. Glucose uptake was measured by the fluorescence intensity of 2-NBDG on *day 9* during differentiation. The results showed that glucose uptake was markedly increased in SVFs from KO mice (Fig. 4, *L* and *M*).

### SMEK1 Affects Glucose Uptake through the AMPK Signaling Pathway

**Figure 5.**
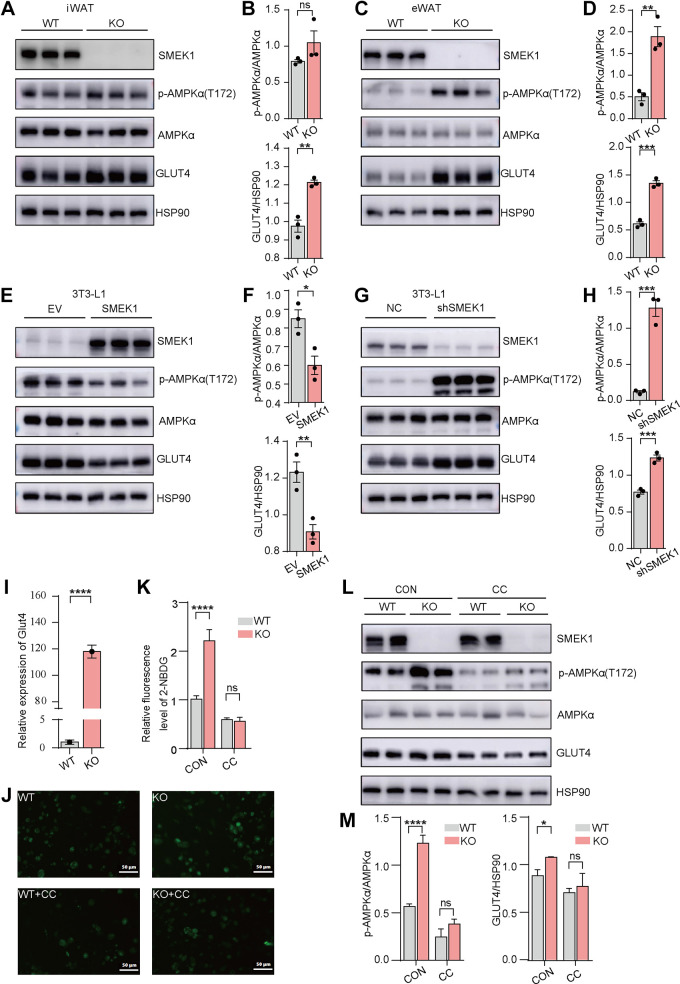
SMEK1 affects glucose uptake through the AMPK signaling pathway. *A*: immunoblotting of p-AMPKα(T172)/AMPKα and GLUT4 protein levels in subcutaneous adipose tissue of WT and KO mice. *B*: quantification of proteins based on A. *C*: immunoblotting of p-AMPKα(T172)/AMPKα and GLUT4 protein levels in epididymal adipose tissue of WT and KO mice. *D*: quantification of proteins based on C. *E*: immunoblotting of p-AMPKα(T172)/AMPKα and GLUT4 protein levels in *Smek1* overexpression and control 3T3-L1 cell lines. *F*: quantification of proteins based on E. *G*: immunoblotting of p-AMPKα(T172)/AMPKα and GLUT4 protein levels in *Smek1* KD and control 3T3-L1 cell lines. *H*: quantification of proteins based on *G*. *I*: the mRNA level of *Glut4* in SVFs isolated from WT and KO mice. *J*: 2-NBDG glucose uptake experiment of SVFs isolated from WT and KO mice using Compound C treatment and control group. Scale bar, 50 μm. *K*: quantification of luminosity based on J. *L*: immunoblotting of p-AMPKα(T172)/AMPKα and GLUT4 proteins in Compound C treated and control groups. *M*: quantification of proteins based on L (ns means no significance, *P < 0.05, **P < 0.01, ***P < 0.001, ****P < 0.0001). GLUT4, glucose transporter 4; KO, knockout; SVFs, stromal-vascular fractions; WT, wild type.

First, we found that compared with their control littermates, the ratio of p-AMPKα(T172)/AMPKα in both iWAT and eWAT from KO mice was increased, while the expression of GLUT4 is also upregulated (Fig. 5, *A* and *C*). The quantification of p-AMPKα(T172)/AMPKα and GLUT4 was measured by Image J software (Fig. 5, *B* and *D*). We induced the differentiation of *Smek1* OE 3T3-L1 cells and *Smek1* KD 3T3-L1 cells into mature adipocytes, then performed immunoblotting of cell samples on *day 9*. As excepted, the ratio of p-AMPKα (T172)/AMPKα and the expression of GLUT4 were reduced in *Smek1* OE 3T3-L1 cells. The quantification was measured by Image J software (Fig. 5, *E* and *F*). On the contrary, the ratio of p-AMPKα(T172)/AMPKα and the expression of GLUT4 are increased in *Smek1* KD 3T3-L1 cells. The quantification was measured by Image J software (Fig. 5, *G* and *H*).

Considering that activation of AMPK can simultaneously enhance *Glut4* transcription and GLUT4 translocation, leading to increased insulin-dependent glucose uptake, we measured the mRNA level of *Glut4.* As mentioned earlier, we isolated SVFs from iWAT of WT or KO mice. When induced differentiation into mature adipocytes on *day 9*, we performed a glucose uptake assay and collected cell samples. The results of real-time quantitative PCR showed that the mRNA level of *Glut4* in *Smek1* KO SVFs is significantly elevated compared with WT SVFs (Fig. 5*I*). Glucose uptake assay was measured by the fluorescence intensity of 2-NBDG. To confirm whether the effects of SMEK1 on glucose uptake are mediated by AMPK activation, we treated adipocytes on *day 9* with Compound C (an AMPK inhibitor). Glucose uptake was markedly increased in SVFs from KO mice, consistent with the results shown in Fig. 4. On the other hand, treatment with Compound C inhibited glucose uptake and ablated the effects of SMEK1 (Fig. 5*J*). The fluorescence intensity of 2-NBDG was measured by Image J software (Fig. 5*K*). We collected cell samples, and the results of immunoblotting confirmed the conclusion. The ratio of p-AMPKα(T172)/AMPKα and the expression of GLUT4 are increased in *Smek1* KO SVFs, while treatment with Compound C inhibited the ratio of p-AMPKα(T172)/AMPKα and the expression of *GLUT4*, and ablated the effects of SMEK1 (Fig. 5*L*). The quantification was measured by Image J software (Fig. 5*M*).

### SMEK1 Ablation Improves Glucose Uptake by Increasing GLUT4 Translocation

**Figure 6.**
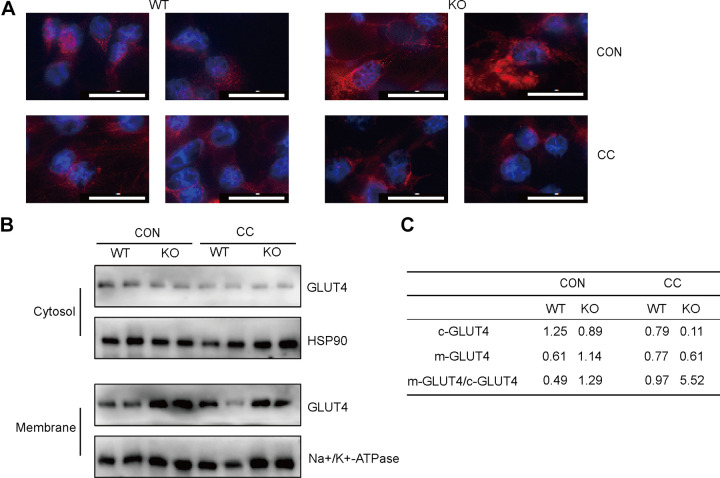
SMEK1 ablation improves glucose uptake by increasing GLUT4 translocation. *A*: representative immunofluorescence staining image of GLUT4. Scale bar, 20 μm. *B*: Western blot analysis of GLUT4 in cytosol (c-GLUT4) and membrane (m-GLUT4). *C*: quantification of proteins based on *B*. GLUT4, glucose transporter 4.

Next, we examined the function of SMEK1 in the translocation of GLUT4 and determined the function of AMPK using Compound C. The translocation of GLUT4 to cell membrane remains the key process of glucose uptake to the intracellular environment. As we predicted, immunofluorescence using GLUT4 antibody conjugated with Alexa fluor 594 was conducted to reveal the increasing amount of embedded GLUT4 in SVFs isolated from *Smek1* KO mice. The increased translocation of GLUT4 to cell membrane by SMEK1 ablation was inhibited by Compound C treatment (Fig. 6*A*). To further investigate the function during the translocation of GLUT4, we separated cell membranes and cytoplasmic proteins of differentiated SVFs. The results of Western blot were consistent with immunofluorescence. The translocation of GLUT4 from cytosol to membrane was markedly increased in SVFs from KO mice, and treatment with Compound C inhibited glucose uptake and ablated the effects of SMEK1(Fig. 6, *B* and *C*). These data suggest that SMEK1 ablation improves glucose uptake by increasing AMPK-regulated GLUT4 translocation.

### SMEK1 Impacts AMPK Signaling Pathway through PPP4C

**Figure 7.**
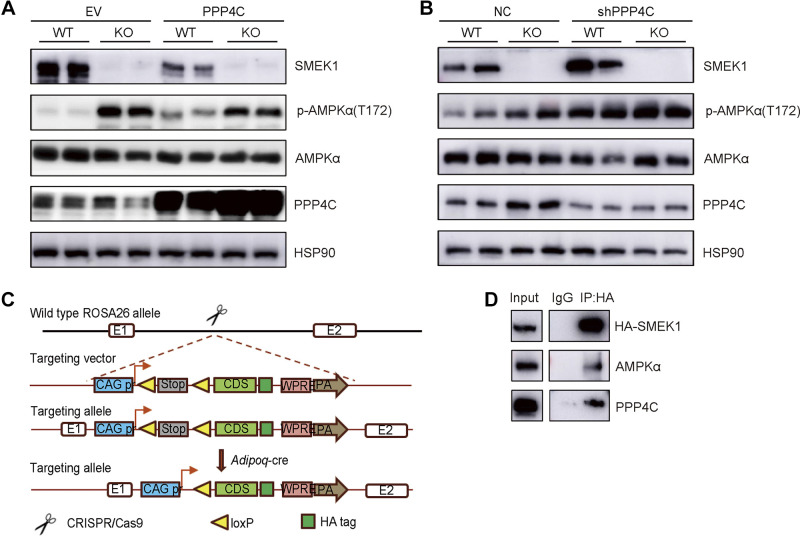
SMEK1 impacts AMPK signaling pathway through PPP4C. *A*: immunoblotting of p-AMPKα(T172)/AMPKα in *PPP4C* overexpression and control SVFs isolated from WT or KO mice. *B*: immunoblotting of p-AMPKα(T172)/AMPKα in *Ppp4c* KD and control SVFs isolated from WT or KO mice. *C*: the construction strategy of *Smek1*-HA mice with specific expression in adipose tissue. *D*: protein immunoprecipitation analysis of the interaction between SMEK1, PPP4C, and AMPKα. KO, knockout; PPP4C, PP4 catalytic subunits; SVFs, stromal-vascular fractions; WT, wild type.

Since SMEK1 has been known as a regulatory subunit of the PP4 enzyme, which might regulate the activity of the PP4 catalytic subunits (PPP4C), we verified the involvement of PPP4C in AMPK dephosphorylation during glucose uptake. First, we overexpressed *PPP4C* using lentivirus carrying *PPP4C*-His vector. Overexpression of *PPP4C* resulted in dephosphorylation of AMPK in SVFs, and the ratio of p-AMPKα(T172)/AMPKα was further downregulated in *Smek1* KO SVFs, demonstrating that PPP4C played a major role in AMPK signaling pathway (Fig. 7*A*). Next, we used the reverse approach to confirm the role of PPP4C. We knocked down *Ppp4c* expression in SVFs isolated from WT or KO mice using lentivirus carrying small hair RNA (shRNA). *Ppp4c* knockdown resulted in restoration of AMPK phosphorylation and ablated the effects of SMEK1 (Fig. 7*B*).

To further confirm our conclusions, we performed immunoprecipitation to identify whether SMEK1, AMPK, and PPP4C exist as a protein complex. As mentioned, we obtained overexpressed *Smek1*-HA in adipose tissue of mice by crossbreeding *ROSA26-Smek1^flox/flox^* mice (21) with *Adipoq*-Cre mice (Fig. 7*C*). We then isolated SVFs from the *Smek1*-HA mice and induced differentiation into mature adipocytes. On *day 9*, we collected cell samples and performed immunoprecipitation to confirm the protein-protein interaction. The assay demonstrated that SMEK1, AMPK, and PP4 bind to one another (Fig. 7*D*). Overall, we proposed a pattern map of the role of SMEK1 in glucose uptake and obesity-related metabolic processes. On the one hand, SMEK1 can inhibit adipogenic differentiation of adipocytes. Under the metabolic pressure of nutrition, it can change the degree of obesity and obesity-related hyperglycemia, hyperlipidemia, insulin resistance. In addition, SMEK1, as a regulatory subunit of PP4, can regulate the ratio of p-AMPKα(T172)/AMPKα through PPP4C. The activation of AMPK affects the expression of *Glut4* mRNA, then changes the glucose uptake capacity of adipocytes.

## DISCUSSION

In this study, we elucidated the important function of SMEK1 in mammalian metabolism. On the one hand, SMEK1 is associated with adipogenic differentiation. An increase in the capacity of adipogenic differentiation following ablation of SMEK1 was observed, confirming the function of SMEK1 in adipogenic differentiation. This may directly affect the obesity of the mice under nutritional stress. On the other hand, SMEK1 also plays an important role in the regulation of glucose homeostasis in vivo or in vitro. The ablation of SMEK1 affects the phosphorylation level of AMPK, directly promotes the transcription of GLUT4, which intuitively alters the capacity of glucose uptake in adipocytes. However, the alteration of p-AMPKα (T172) is most likely due to the direct effect of PPP4C.

In many previous studies, SMEK1 existed as a regulatory subunit of PP4. PP4 is an important protein phosphatase that can exert a range of important functions through dephosphorylation of downstream proteins. These include DNA damage repair, genome stability, immune responses (22), and glucose homeostasis (9). Research on PP4 initially focused mostly on PPP4C, and later some studies began to show that although the catalytic subunit PPP4C did not change, binding to different regulatory subunits can alter the activity, substrate specificity, subcellular location of the holoenzyme (12). Therefore, in recent years, studies involving regulatory subunits (PPP4R1, PPP4R2, PPP4R3 and so on) have been increasing in number.

As we mentioned earlier, the biological functions of SMEK1 involve various aspects of neuronal differentiation and development (23, 24), cortical neurogenesis (25), histone deacetylation (26), antitumor processes (27, 28), miRNA biosynthesis (29), and transcription initiation (30). In 2010, Yoon et al. reported that SMEK/PP4C protein was involved in the regulation of hepatic gluconeogenesis by reducing the phosphorylation level of CRTC2 (14). In 2014, Ma et al reported that Pph3-Psy2, a homologue of human SMEK1/PP4C in yeast, affects the expression of glucose transporter genes by dephosphorylating MTH1 (15). In 2023, Huang et al. identified that SMEK1 played a more extensive metabolic function. When lipids, arabinose, or ethanol were used as a carbon source, SMEK1 enhanced the expression of genes involved in fatty acid catabolism, glyoxylate cycle, methyl citrate, tricarboxylic acid cycle, ethanol metabolism by activating the transcription activator CRF1. When glucose was used as a carbon source, SMEK1 activated the transcriptional repressor CREA, which inhibited the expression of glyoxylate cycle, tricarboxylic acid cycle, lipolysis, fatty acid catabolism, and methyl citrate cycle. Although the regulation of CREA or CRF1 activity by SMEK1 is based on its dephosphorylation, no final elucidation of whether the dephosphorylation of SMEK1 is mediated through PP4C was made in this article. But in a model of △crf1 (lipid metabolism and arabinose utilization are known altered), SMEK1, but not PP4C, was identified as a differential gene, suggesting that SMEK1 acts as a regulatory subunit of PP4 and may have an independent biological function (11).

Given previous studies on PP4 and SMEK1, we first hypothesized that SMEK1 plays an important role in glucose homeostasis. To verify our hypothesis, also sought additional experimental clues, we performed a correlation analysis of SNP loci localized in SMEK1 with metabolic phenotypes in the GWAS database (31). As we expected, the results showed that among the metabolic phenotypes associated with SMEK1, the strongest association appeared to be glucose homeostasis (including fasting serum glucose, glucose tolerance-related). In addition, some SNPs of SMEK1 were also associated with diabetes, insulin homeostasis, body weight, and other phenotypes. This prompted us to investigate the role of SMEK1 in obesity and glucose homeostasis.

First, we found that SMEK1 is highly expressed in white adipose tissues of individuals with obesity. Mice model, datasets, as well as our collected human samples concur with this conclusion. Analysis of the GEPIA database and experiments in vitro and in vivo demonstrate the role of SMEK1 in adipogenesis. Overall, adipogenesis now emerges as a viable therapeutic target and is gaining attention for its potential application in the clinic (3, 4, 32).

We know that adipose tissue expands in two ways, either by differentiating to produce more adipocytes or by increasing the size of adipocytes (33). Change in adipocyte size is associated with lipid synthesis and lipolysis. In fact, the results from the GEPIA database also suggest that there is a significant correlation between SMEK1 and genes related to lipolysis (*PLIN1*, *HSL* and *ATGL*) both in iWAT and eWAT (Supplemental Fig. S2). This is in line with what we mentioned earlier, SMEK1 regulates lipolysis during infection by *Magnaporthe*. Here we suggest that SMEK1 may play a dual role in adipogenic differentiation and lipolysis, which could explain the phenotype of reduced adipocytes and adipose tissue in KO mice, as well as the healthier metabolic status. Of course, since we used *Smek1* KO mice in our experiment, existing studies have shown that genes ablation in brain, neurons, liver, gut, skeletal muscle, and macrophages also can alter adipose tissue and systemic metabolic homeostasis (34–37). So even if we are certain about the role of SMEK1 in adipocytes, we did not exclude that the phenotype of *Smek1* KO mice is due to the role in other tissues and organs, so we will further investigate the role of SMEK1 by mice with adipose tissue-specific knockout of *Smek1*.

Obesity tends to promote insulin resistance, and our results confirmed it. It is worth mentioning that our study also found differences in fasting blood glucose levels, which is quite reasonable because when insulin signaling is impaired, hepatocytes and adipocytes cannot use glucose efficiently (38, 39). However, we prefer that SMEK1 has a direct role in the glucose uptake process, suggesting that AMPK is a definite intermediate factor, its phosphorylation is indeed closely related to both SMEK1 and glucose uptake mediated by GLUT4.

Although the level of fasting blood glucose level was lower in KO mice, they have an enhanced ability of gluconeogenesis. This result may be due to several reasons. Glucagon action in the liver is to promote glucose output through gluconeogenesis and the basal glucagon level in fasting accounts for up to 70% of glucose production. We found an increased level of glucagon in KO mice. Glucagon-induced hepatic glucose production may result in enhanced gluconeogenesis. In addition to pyruvate, products of fat breakdown such as glycerol also serve as raw materials for gluconeogenesis. We observed an increase in lipolysis under SMEK1 ablation. Although we did not delve into more details due to the topic of article, theoretically, KO mice should have higher levels of glycerol in serum, which may lead to activation of gluconeogenesis. The specific mechanism depends on further research in other organs like liver, which we did not cover.

With regard to the effect of SMEK1 on the phosphorylation of AMPK, we still believe that it is related to the effect of PP4C in the holoenzyme. SMEK1, PP4C, and AMPK indeed exist as a complex in mature adipocytes. We also found that the effect of SMEK1 ablation in adipocytes was not completely abolished after the knockdown or overexpression of *PPP4C*, possibly due to the nonnegligible effect of regulatory subunits on holoenzyme. In addition, we found that PPP4C was frequently decreased in the tissue of KO mice, suggesting that regulatory subunits in the holoenzyme may also affect the expression of catalytic subunits. In addition, we do not rule out that SMEK1 has a metabolic function independent of PP4, which still needs more in-depth studies to prove.

In conclusion, our findings contributed to the current understanding of adipogenesis and glucose homeostasis, identified new possibilities for the treatment of obesity-related metabolic disorders.

## DATA AVAILABILITY

Data will be made available on request.

## SUPPLEMENTAL DATA

10.6084/m9.figshare.25466872 Supplemental Figs. S1–S3: http://doi.org/10.6084/m9.figshare.25466872.
Supplemental Tables S1–S3: http://doi.org/10.6084/m9.figshare.25466809.

## GRANTS

This work was supported by grants from the National Key R&D Program of China (No. 2022YFC2703701 to Q.L.) and the National Natural Science Foundation of China (No. 82271901, 32070586).

## DISCLOSURES

No conflicts of interest, financial or otherwise, are declared by the authors.

## AUTHOR CONTRIBUTIONS

S.W. and Q.L. conceived and designed research; S.W., Y.S., Z.L., and Z.W. performed experiments; S.W., Y.S., and Q.L. analyzed data; S.W., A.L., Y.X., Y.J., W.S., and J.L. interpreted results of experiments; S.W., S.G., and H.S. prepared figures; X.L. drafted manuscript; P.S. edited and revised manuscript; Q.L. approved final version of manuscript.
